# Searching for novel cell cycle regulators in *Trypanosoma brucei *with an RNA interference screen

**DOI:** 10.1186/1756-0500-2-46

**Published:** 2009-03-23

**Authors:** Séverine Monnerat, Caroline Clucas, Elaine Brown, Jeremy C Mottram, Tansy C Hammarton

**Affiliations:** 1 Division of Infection & Immunity, Faculty of Biomedical and Life Sciences and Wellcome Centre for Molecular Parasitology, University of Glasgow, Glasgow, UK

## Abstract

**Background:**

The protozoan parasite, *Trypanosoma brucei*, is spread by the tsetse fly and causes Human African Trypanosomiasis. Its cell cycle is complex and not fully understood at the molecular level. The *T. brucei *genome contains over 6000 protein coding genes with >50% having no predicted function. A small scale RNA interference (RNAi) screen was carried out in *Trypanosoma brucei *to evaluate the prospects for identifying novel cycle regulators.

**Results:**

Procyclic form *T. brucei *were transfected with a genomic RNAi library and 204 clones isolated. However, only 76 RNAi clones were found to target a protein coding gene of potential interest. These clones were screened for defects in proliferation and cell cycle progression following RNAi induction. Sixteen clones exhibited proliferation defects upon RNAi induction, with eight clones displaying potential cell cycle defects. To confirm the phenotypes, new RNAi cell lines were generated and characterised for five genes targeted in these clones. While we confirmed that the targeted genes are essential for proliferation, we were unable to unambiguously classify them as cell cycle regulators.

**Conclusion:**

Our study identified genes essential for proliferation, but did not, as hoped, identify novel cell cycle regulators. Screening of the RNAi library for essential genes was extremely labour-intensive, which was compounded by the suboptimal quality of the library. For such a screening method to be viable for a large scale or genome wide screen, a new, significantly improved RNAi library will be required, and automated phenotyping approaches will need to be incorporated.

## Background

The *Trypanosoma brucei *cell cycle is complex and is regulated differently in the mammalian bloodstream and insect procyclic life cycle stages. Its regulation also diverges from mammalian cell cycle regulation, suggesting that some of its regulators might have potential as novel drug targets [1]. Many cell cycle regulators in *T. brucei *remain unidentified, not least because 56% of genes in the genome are currently annotated as hypothetical open reading frames (ORFs) . RNAi screens have previously been used to identify cell cycle regulators in model organisms [2-8], and to identify essential genes on chromosome I in bloodstream stage (BS) *T. brucei *[9]. In addition, use of a *T. brucei *RNAi genomic library has identified a hexokinase that modulates procyclin expression [10] and a protein p166 involved in kinetoplast DNA replication [11]. We hypothesised that screening this library would allow us to identify essential novel cell cycle regulators.

### Approach

Procyclic form (PF) parasites were screened because of their greater transformation efficiency compared to BS parasites. The cell line 427 pLew13 pLew29 [12] was transfected [13] with the RNAi library [10] and 204 independent clones were selected by limiting dilution. Clones were characterised individually to identify those displaying proliferation defects following RNAi induction with tetracycline, and RNAi library inserts sequenced to identify the targeted gene. Clones targeting a protein coding gene and showing a proliferation defect were characterised for cell cycle defects using flow cytometry and DAPI staining analyses [14]. Where potential cell cycle defects were identified, new RNAi cell lines were generated and the analysis repeated in an attempt to confirm the original phenotype in the PF and to determine whether these genes were involved in cell cycle regulation in BS trypanosomes.

## Results

### Identification of RNAi library inserts

RNAi library vector inserts (integrated into the rDNA spacer region of the genome) were PCR-amplified from genomic DNA of clones, sequenced and analysed by BLAST analysis at GeneDB  or NCBI . Sequence data was only obtained for 155 clones, but showed them to be unique [see Additional file 1]. For the rest, either the PCR or the sequencing failed. Some library plasmids may have contained no insert, but technical issues relating to the lack of standard sequencing primer binding sites within the RNAi plasmid may have also contributed. Of the 155 sequenced inserts, 52 contained sequences of no interest for this screen (eg *VSG*/*ESAG *genes, repeat regions, intergenic sequences outside of UTRs etc) and a further 25 inserts could not be identified by BLAST, which, since the library was made from total genomic DNA, could have come from intermediate or mini-chromosomes that were not sequenced in the *T. brucei *genome project [15]. Hence, about 60% clones obtained using this library were of no practical use for identifying the essential cell cycle regulators we sought. It is also worth noting that 18 clones deemed to be of no practical use nevertheless showed a proliferation defect following RNAi induction [see Additional file 1], but we did not study these clones further.

Of the remaining clones, 17 contained sequence from known, non-*VSG/ESAG*, genes and 36 represented hypothetical genes. Some targeted 5' or 3' UTRs rather than the ORF itself. A further 17 inserts spanned over 2 genes, and for 8 clones, two PCR products were obtained.

### Initial screening

Sixteen of the 76 clones targeting non-VSG/ESAG protein-coding genes gave proliferation defects following RNAi induction [see Additional file 2] and [Additional file 3]. Two of these targeted previously studied essential genes: radial spoke protein 3, *RSP3*, (clone 33) [16] and a member of the exosome complex, *RRP44 *(clone 45) [17], validating our primary screen. Twelve clones [see Additional file 2] and a negative control clone were analysed further (secondary screening). Growth curves were repeated to confirm proliferation defects and cell cycle progression was monitored [14] [see Additional file 4]. As expected, no defects occurred upon induction of the negative control (clone 165). Clone 33 (targeting RSP3 [16]) acted as a positive control and upon induction, displayed proliferation and cell cycle defects, consistent with previously published data [see Additional file 4]. Clone 45 (RRP44 [17]) proliferated poorly in the secondary screen, displaying cell cycle defects even when non-induced, suggesting leaky expression from the RNAi vector [see Additional file 4] [18]. Since RRP44 is required for rRNA processing [17], its depletion is likely to result in pleiotropic effects on the cell, and hence the cell cycle defects probably occur indirectly.

For six clones (8, 13, 44, 174, 209 and 211), RNAi induction confirmed proliferation defects from the primary screen (except for clone 13, which grew poorly in the absence of induction) and also revealed cell cycle defects [see Additional file 4]. Four clones targeted genes of potential importance for cell cycle progression: clones 8 and 211 (TOR-like 2 and TOR1 kinases, respectively [19]), clone 13 (putative protein phosphatase 1-like (PP1)) and clone 209 (hypothetical ORF). The cell cycle defects observed with clone 174 (dynein heavy chain) were likely to reflect flagellar motility defects, but as this particular gene had not been studied previously, it was included in subsequent analyses. Induction of clone 44, targeting an electron transfer protein, probably also caused indirect effects on the cell cycle, and was not analysed further. The remaining clones were eliminated since either the previously observed proliferation defects were not reproduced (clones 135, 153 and 223), or despite the targeted genes being essential for viability, no cell cycle defects were observed (clone 187, targeting two conserved hypothetical genes).

### Tertiary screen

**Figure 1:**
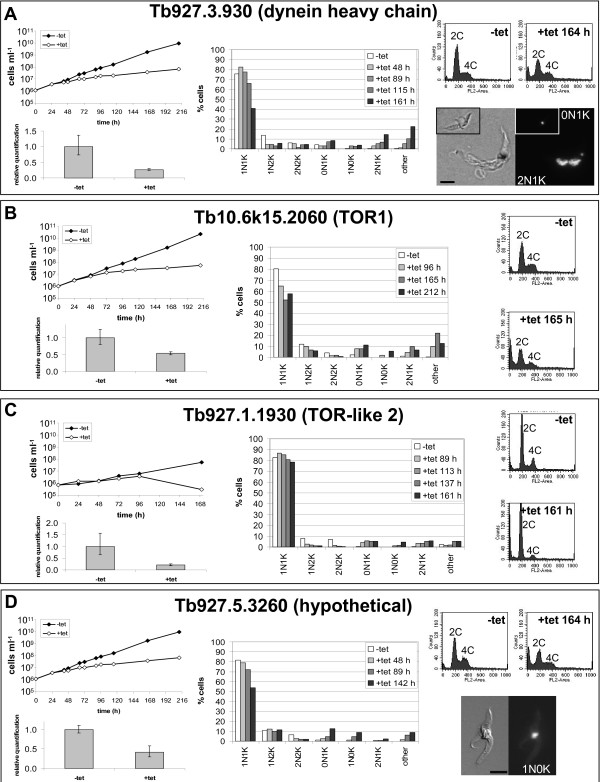
**Tertiary screening in the procyclic form**. Independent RNAi cell lines generated in PF *T. brucei *for dynein heavy chain (Tb927.3.930), panel A; TOR1 (Tb10.6k15.2060), panel B; TOR-like 2 kinase (Tb927.1.1930), panel C and a hypothetical ORF (Tb927.5.3260), panel D, were analysed for cell cycle progression defects following RNAi induction. Upper left of each panel: cumulative growth curves for each clone cultured in SDM79 with appropriate selective drugs [13,14] and the absence (-tet) or presence (+tet) of 1 μgml^-1 ^tetracycline. Lower left of each panel: real time PCR analysis of mRNA levels for each gene (using oligonucleotides detailed in Additional file 5 and standardised against a *GPI8 *control) at 96 hrs (dynein heavy chain and hypothetical ORF), 161 hrs (TOR-like 2 kinase) or 212 hrs (TOR1) post-induction. Middle of each panel: analysis of nuclei and kinetoplast numbers as determined over time by DAPI staining. Upper right of panels A and D and right of panels B and C: flow cytometry profiles of uninduced (-tet) and induced (+tet) cells at the time points indicated. The DNA content of each peak is given. Lower right panels A and D: examples of abnormal cells generated. Left, DIC image; right, DAPI image. Black bar: 5 μm. The number of nuclei (N) and kinetoplasts (K) in each cell is given.

**Figure 2:**
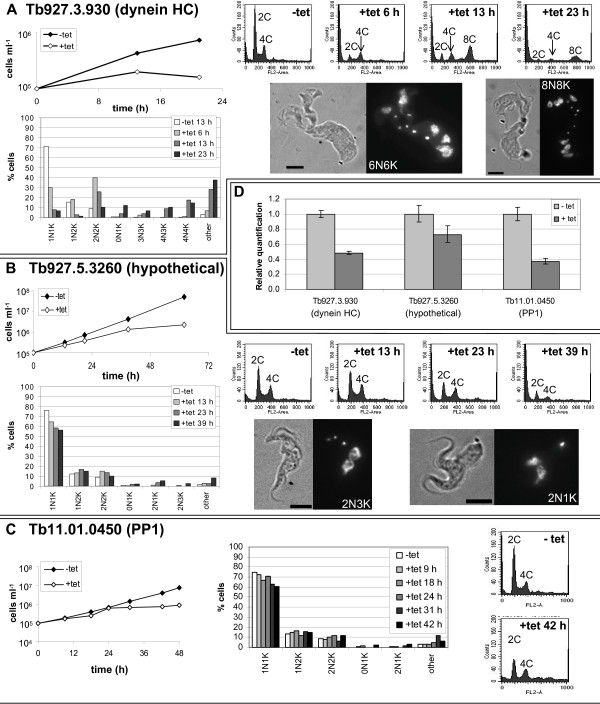
**Tertiary screening in the bloodstream stage**. Analysis of RNAi cell lines generated in BS *T. brucei *for dynein heavy chain (Tb927.3.930, Panel A), hypothetical ORF (Tb927.5.3260, Panel B) and protein phosphatase 1 (Tb11.01.0450, Panel C). Panels A and B, upper left and Panel C, left: cumulative growth curves for each clone cultured in HMI9 with appropriate selective drugs [13,14] in the absence (-tet) or presence (+tet) of tetracycline; Panels A and B, lower left and Panel C, middle: analysis of nuclei and kinetoplast numbers as determined over time by DAPI staining; Panels A and B, upper right and Panel C, right: flow cytometry profiles at the time points indicated; Panels A and B, lower right: examples of abnormal cells generated (left, DIC image; right, DAPI image. Black bar: 5 μm. The number of nuclei (N) and kinetoplasts (K) in each cell is given). Panel D: real time PCR analysis of mRNA levels for each gene (standardised against a *GPI8 *endogenous control, using oligonucleotides detailed in Additional file 5) at 6.5 hrs (dynein heavy chain) and 16 hrs (hypothetical ORF and PP1) post-induction.

To confirm phenotypes observed for each target, a gene-specific DNA fragment (checked for suitability for RNAi using RNAit [20]) was cloned into the vector p2T7^ti ^[21] ([see Additional file 5] for oligonucleotide details), before being transfected into PF (427 pLew13 pLew29) and, where appropriate, BS (427 pLew13 pLew90) cell lines [12]. RNAi of TOR1 and TOR-like 2 kinases in BS trypanosomes have been described elsewhere [9,19]. For *PP1*, although we isolated BS clones, no PF transformants were obtained, despite repeated attempts. Depletion of mRNA following RNAi induction was confirmed by real time PCR analysis (Figs. 1 and 2D), and resulted in reduced proliferation rates in the PF (Fig. 1) and BS (Fig. 2).

### The dynein heavy chain Tb927.3.930 is essential for motility and cell cycle progression

Downregulation of Tb927.3.930 caused a significant reduction in motility (not shown), accompanied by significant cell cycle changes. In the PF, cells with abnormal complements of nuclei (N) and kinetoplasts (K), including 0N1K, 2N1K and >2N2K, were observed (Fig. 1A). The 2N1K cells could have arisen following an aberrant cytokinesis event (e.g. 2N2K cell dividing to give 2N1K + 0N1K daughter cells) or because of defective flagellar/basal body/kinetoplast replication or segregation. Most multi-nucleate cells contained fewer kinetoplasts than nuclei, also suggesting impeded kinetoplast re-replication/segregation. In the BS, the proportion of 2N2K cells increased from 9% to 40% over the first 6 hours of induction (Fig. 2A). Less than 10% of these cells were observed to be furrowing or undergoing abscission (not shown). At later time points, these cells re-replicated their DNA, leading to the appearance of cells with 8C DNA content and cells containing >2N2K. Approximately half of these cells had now undergone furrowing and were arrested at abscission, in some cases, with multiple cell bodies (Fig. 2A).

Despite the cell cycle defects observed, dynein is unlikely to regulate the cell cycle directly. Dyneins, comprised of heavy, intermediate, light intermediate and light chains, are motor proteins that, in the flagellum, generate the force required for motility, which is known to be essential for cytokinesis in BS trypanosomes [22]. The dynein heavy chain targeted here is an outer arm dynein-α heavy chain [23] that was detected in a *T. brucei *flagellum proteome [22]. Previously, RNAi of the dynein intermediate chain, DNAI1, in procyclic *T. brucei *resulted in cells with axonemes lacking outer dynein arms that no longer displayed forward motility [24]. Downregulation of the dynein light chain, LC1, lead to procyclic trypanosomes displaying a jerky swimming pattern, but attempts to downregulate a dynein heavy chain, DNAH, failed [24]. Our data are therefore consistent with, and extend, previous functional data on axonemal components.

### Depletion of two TOR family kinases disrupts the procyclic cell cycle

Following RNAi of *TOR1 *in procyclic *T. brucei*, 2N2K cells were almost abolished (Fig. 1B) and 2N1K, 0N1K and other abnormal cell types appeared, indicating disruption to cell cycle progression. However, since these cell types can arise in many ways, further analysis will be required to establish the origin of these cells and to determine if TOR1 is really a direct regulator of the cell cycle. In bloodstream trypanosomes, depletion of TOR1 decreases protein synthesis, giving rise to smaller sized cells that accumulate in G_1 _phase [19]. At first glance, TOR1 depletion in procyclic parasites seems to cause very different effects from those reported for bloodstream trypanosomes and clearly warrants further investigation.

Downregulation of TOR-like 2 in the PF abolished 1N2K cells, which could indicate defects in kinetoplast replication or segregation (Fig. 1C). This is supported by the reduction of 2N2K cells and concomitant appearance of 2N1K cells. However, other abnormal cell types (0N1K, multinucleate cells) were also observed, and as above, further analysis is required before TOR-like 2 can be classified as a cell cycle regulator. RNAi of TOR-like 2 has previously been performed in BS trypanosomes, but no phenotypes were observed [9].

### The hypothetical ORF, Tb927.5.3260, and PP1 (Tb11.01.0450) are essential for proliferation but may not be required for cell cycle control

In PF parasites, RNAi of the hypothetical ORF (Tb927.5.3260) caused changes to the cell cycle profile (Fig. 1D), but these defects only accumulated in significant numbers at late time points, suggesting that they could be downstream effects of another defect. In the BS, only subtle changes in cell cycle profile were observed following RNAi induction (Fig. 2B). Hence, at present, we cannot conclude that this protein plays a role in cell cycle control. However, given that it is essential for proliferation and there are orthologues in *Leishmania major *and *Trypanosoma cruzi*, its role warrants further investigation. RNAi of PP1 also did not lead to significant cell cycle changes in BS trypanosomes (Fig. 2C), and therefore, although it is apparently essential for proliferation, it may not regulate the cell cycle. Previously, depletion of all seven PP1 genes simultaneously in PF trypanosomes, reduced proliferation but did not effect the cell cycle [25], although okadaic acid (a PP1 and PP2A inhibitor) treatment disrupts kinetoplast segregation [26].

## Conclusion

We performed an RNAi screen to determine the feasibility of genome-wide screening for *T. brucei *cell cycle regulators. We identified genes (mostly previously unstudied) essential for PF growth. However, we could not demonstrate any to be direct regulators of the cell cycle. Known cell cycle regulators such as cyclins and cyclin-dependent kinases were not identified, although this is likely to be due to an issue of coverage. There are only 21 cyclin and CDKs in *T. brucei *[1,27], and the 76 clones analysed in this screen target <1% of the protein coding genes in *T. brucei*. The screen itself was highly inefficient. Despite isolating over 200 independent clones, only 76 (38%) (excluding clones that targeted *VSG/ESAG *genes) were confirmed by sequence analysis to target protein coding genes. Although this would not cause too many problems if carrying out a positive selection screen (eg using pooled RNAi clones to look for non-essential phenotypes such as lack of concanavalin A binding [28]), it was a significant issue here, where clones were screened individually for an essential phenotype. Significant time was expended in generating, culturing and analysing clones, which later turned out to be of no interest. To efficiently screen for essential genes in the future using a forward genetics approach, a new RNAi library will be required. As a minimum, this library should be megabase chromosome-specific, lack highly repetitive sequences and contain standard sequencing primer binding sites. Ideally it would also be restricted to containing fragments of protein-coding genes only, would lack intergenic sequences, and for the majority of assays, it would be preferable for it to lack *VSG/ESAG *gene sequences. For a large scale or whole genome screen, it would also be necessary to automate the DAPI staining analysis using, for example, high content microscopy technology.

## Abbreviations

RNAi: RNA interference; N: nucleus; K: kinetoplast; ORF: open reading frame; PP1: protein phosphatase 1; PF: procyclic form; BS: bloodstream stage.

## Competing interests

The authors declare that they have no competing interests.

## Authors' contributions

SM, CC and EB generated RNAi clones and carried out the primary screen (growth rate analysis). Secondary screening (subsequent cell cycle analysis of clones) was carried out by SM and CC, with some assistance from TH, while tertiary screening in procyclic and BS parasites was performed primarily by SM and TH. JM and TH conceived of the study and participated in its design and coordination. TH wrote the manuscript with help from JM, SM and CC. All authors read and approved the final manuscript.

## Supplementary Material

Additional File 1 RNAi vector inserts (integrated into the ribosomal DNA spacer region) were PCR-amplified from genomic DNA of procyclic clones analysed in this study, sequenced and analysed by BLAST. Click here for file

Additional File 2 Details of clones targeting non-VSG/ESAG protein coding genes, displaying a proliferation defect upon induction are given. The twelve clones for which secondary screening was performed, as well as the negative control clone 165, are highlighted in grey. Click here for file

Additional File 3 Procyclic library clones were cultured [13] in the absence (-tet) or presence (+tet) of tetracycline for 216 hours (9 days), and cells were counted every 48–72 hours using a Coulter counter. Representative cumulative growth curves for different phenotypic classes of RNAi library clones are shown. A: no growth defect; B: growth arrest, C: slow growth defect, following RNAi induction. The identities of RNAi clones are given for each graph. Growth curves for all clones tested can be found at . Click here for file

Additional File 4 Selected procyclic library clones were cultured in the absence (-tet) or presence (+tet) of tetracycline. Cell densities were determined daily using a Neubauer Improved haemocytometer and phenotype analysis was carried out at appropriate time points. Cumulative growth curves (left), flow cytometry profiles at the time points indicated (middle) and nucleus/kinetoplast configurations as determined by DAPI staining (right) are shown. Data for the negative control clone sGL165 is included for comparison. Click here for file

Additional File 5 The sequences, targets and properties of the oligonucleotides used in this study are given. Click here for file
