# Supplementary material for: Searching for novel cell cycle regulators in Trypanosoma brucei with an RNA interference screen
Source: BMC Res Notes. 2009 Mar 23;2:46. doi: 10.1186/1756-0500-2-46 (PMC2674452; doi:10.1186/1756-0500-2-46)
Supplement: Additional File 2 — Details of clones targeting non-VSG/ESAG protein coding genes, displaying a proliferation defect upon induction are given. The twelve clones for which secondary screening was performed, as well as the negative control clone 165, are highlighted in grey. [file 1756-0500-2-46-S2.doc]

|  | **Clone** | **Hour effect first detected from** | **Insert ID** | **GeneDB identifier** |
| --- | --- | --- | --- | --- |
| **Growth arrest** | 44 | 96 | Electron transfer protein | Tb927.8.3380 |
|  | 187 | 144 | Insert spans two conserved hypothetical genes | Tb09.211.1940/  Tb09.211.1950 |
|  | | | | |
| **Slow growth defect** | 8 | 96 | TOR-like 2 kinase | Tb927.1.1930 |
|  | 11 | 96 | Conserved hypothetical | Tb927.8.7490 |
|  | 12 | 96 | Insert spans two conserved hypothetical genes | Tb11.12.0004/  Tb11.12.0005 |
|  | 13 | 96 | Protein phosphatase 1 | Tb11.01.0450 |
|  | 24 | 144 | Conserved hypothetical | Tb11.01.8690 |
|  | 33 | 96 | RSP3 | Tb11.47.0034 |
|  | 38 | 96 | Hypothetical | Tb927.1.2650 |
|  | 45 | 96 | RRP44 (exosome complex) | Tb11.01.0260 |
|  | 135 | 144 | Protein kinase | Tb11.02.3010 |
|  | 153 | 72 | 2 PCR products amplified – intergenic sequence and ubiquinol cytochrome C reductase | Tb927.4.4990 or Tb927.8.7430 |
|  | 174 | 72 | Dynein heavy chain | Tb927.3.930 |
|  | 209 | 72 | Conserved hypothetical | Tb927.5.3260 |
|  | 211 | 72 | TOR1 | Tb10.6k15.2060 |
|  | 223 | 144 | Conserved hypothetical | Tb927.2.6080 |
|  | | | | |
| **No growth defect** | 165 |  | Conserved hypothetical | Tb10.70.3090 |
