# Supplementary figures and images for: Searching for novel cell cycle regulators in Trypanosoma brucei with an RNA interference screen
Source: BMC Res Notes. 2009 Mar 23;2:46. doi: 10.1186/1756-0500-2-46 (PMC2674452; doi:10.1186/1756-0500-2-46)

## Slide 1
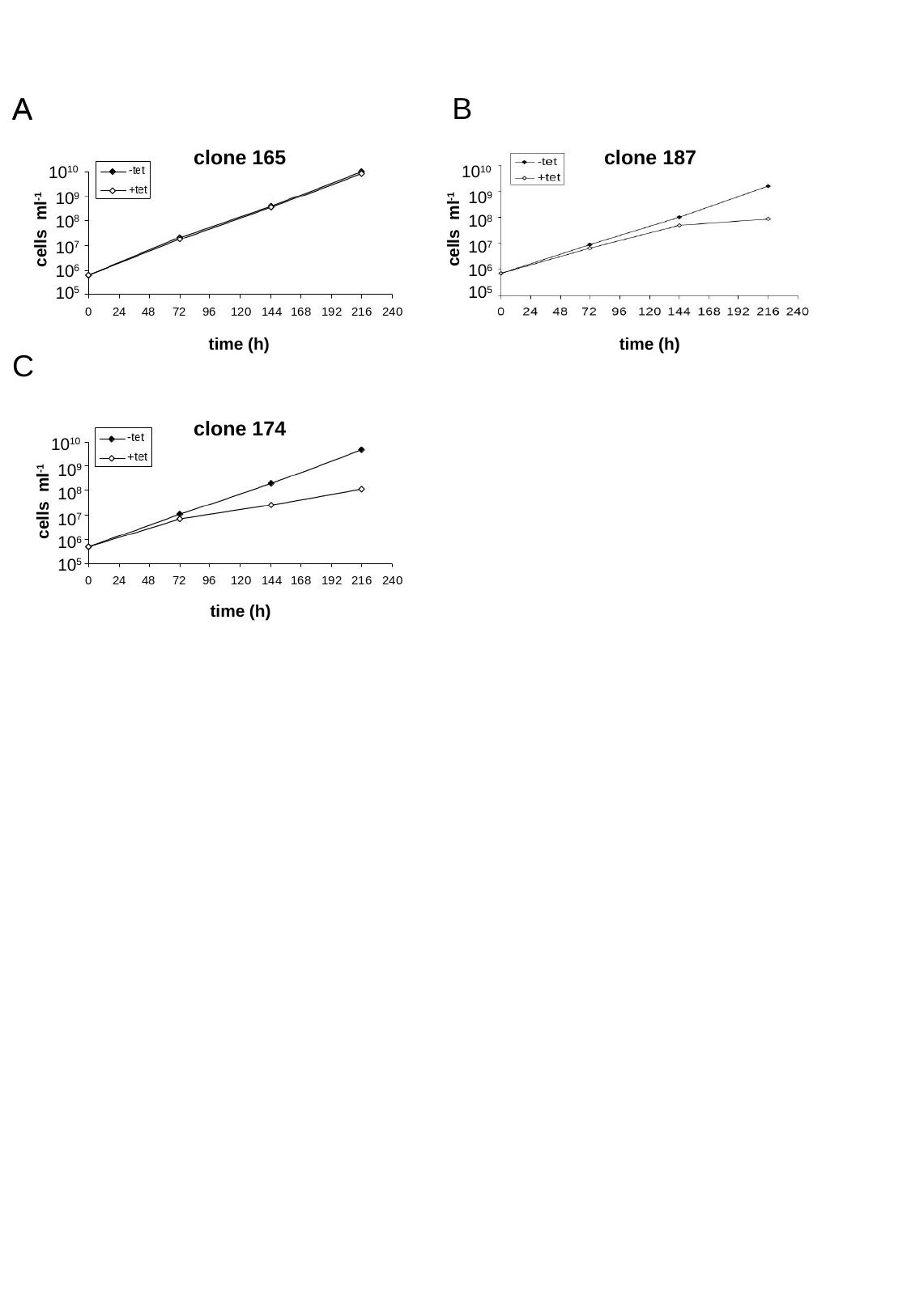

A
B
A
clone 165
clone 187
1010
109
108
cells ml-1
107
106
105
1010
109
108
cells ml-1
107
106
105
time (h)
time (h)
C
clone 174
1010
109
108
cells ml-1
107
106
105
time (h)

Supplement: Additional File 3 — Procyclic library clones were cultured [13] in the absence (-tet) or presence (+tet) of tetracycline for 216 hours (9 days), and cells were counted every 48–72 hours using a Coulter counter. Representative cumulative growth curves for different phenotypic classes of RNAi library clones are shown. A: no growth defect; B: growth arrest, C: slow growth defect, following RNAi induction. The identities of RNAi clones are given for each graph. Growth curves for all clones tested can be found at . [file 1756-0500-2-46-S3.ppt]
