# Supplementary material for: Searching for novel cell cycle regulators in Trypanosoma brucei with an RNA interference screen
Source: BMC Res Notes. 2009 Mar 23;2:46. doi: 10.1186/1756-0500-2-46 (PMC2674452; doi:10.1186/1756-0500-2-46)
Supplement: Additional File 4 — Selected procyclic library clones were cultured in the absence (-tet) or presence (+tet) of tetracycline. Cell densities were determined daily using a Neubauer Improved haemocytometer and phenotype analysis was carried out at appropriate time points. Cumulative growth curves (left), flow cytometry profiles at the time points indicated (middle) and nucleus/kinetoplast configurations as determined by DAPI staining (right) are shown. Data for the negative control clone sGL165 is included for comparison. [file 1756-0500-2-46-S4.ppt]

## Slide 1
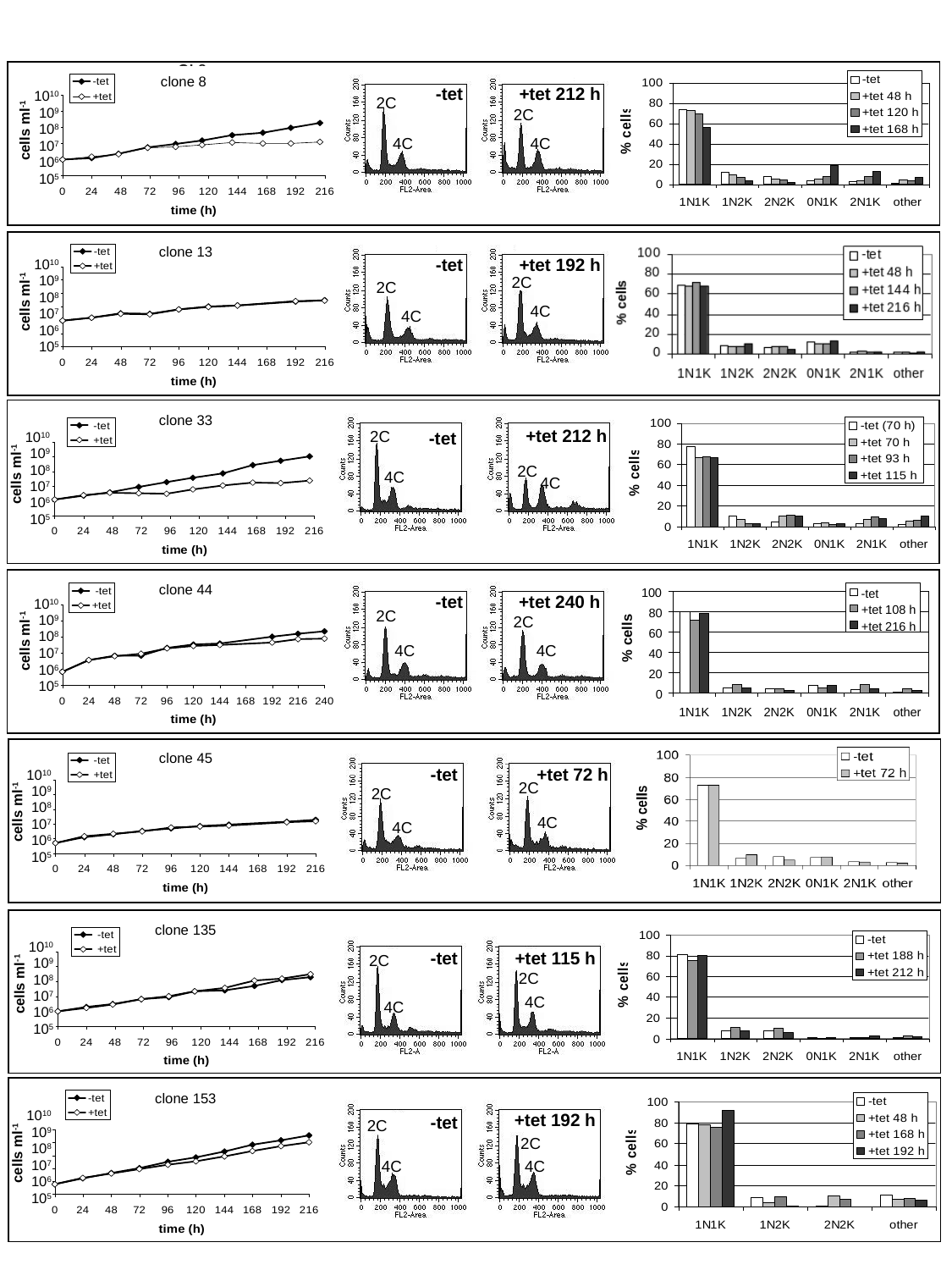

1010
109
108
cells ml-1
107
106
105
clone 8
-tet
+tet 212 h
2C
2C
4C
4C
1010
109
108
cells ml-1
107
106
105
clone 13
-tet
+tet 192 h
2C
2C
4C
4C
1010
109
108
cells ml-1
107
106
105
2C
4C
2C
4C
+tet 212 h
-tet
clone 33
1010
109
108
cells ml-1
107
106
105
-tet
+tet 240 h
2C
2C
4C
4C
100
-tet
+tet 108 h
80
+tet 216 h
60
% cells
40
20
0
1N1K
1N2K
2N2K
0N1K
2N1K
other
clone 44
1010
109
108
cells ml-1
107
106
105
2C
4C
2C
4C
-tet
+tet 72 h
clone 45
1010
-tet
+tet 115 h
2C
109
2C
108
cells ml-1
107
4C
4C
106
105
clone 135
1010
109
108
cells ml-1
107
106
105
+tet 192 h
-tet
2C
2C
4C
4C
clone 153

## Slide 2
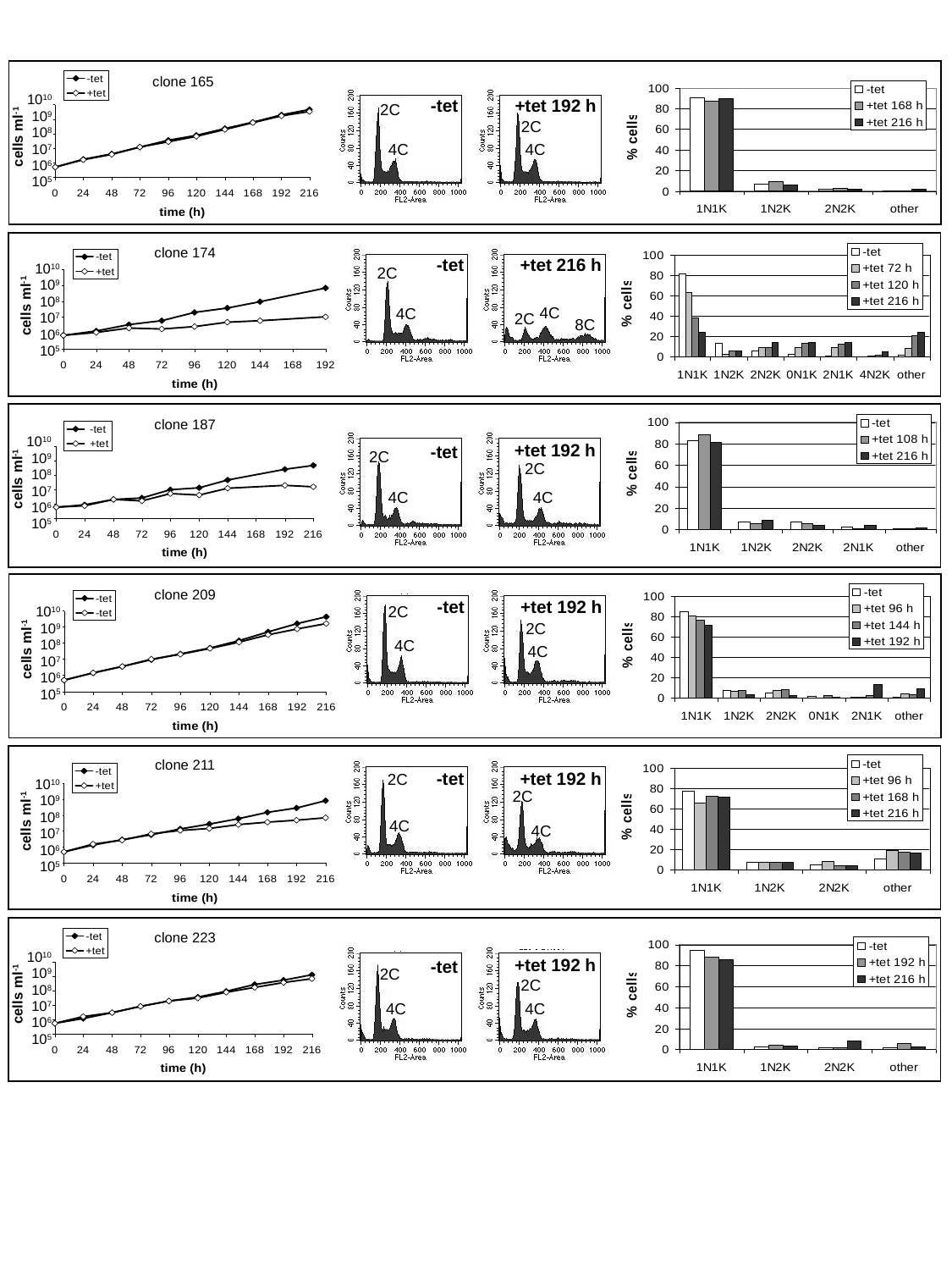

1010
109
108
cells ml-1
107
106
105
2C
4C
2C
4C
-tet
+tet 192 h
clone 165
1010
109
108
cells ml-1
107
106
105
-tet
+tet 216 h
2C
4C
4C
2C
8C
clone 174
1010
109
108
cells ml-1
107
106
105
2C
4C
2C
4C
+tet 192 h
-tet
clone 187
1010
109
108
cells ml-1
107
106
105
-tet
+tet 192 h
2C
2C
4C
4C
clone 209
1010
109
108
cells ml-1
107
106
105
-tet
+tet 192 h
2C
2C
4C
4C
clone 211
1010
109
108
cells ml-1
107
106
105
+tet 192 h
-tet
2C
2C
4C
4C
clone 223
