# Supplementary material for: Searching for novel cell cycle regulators in Trypanosoma brucei with an RNA interference screen
Source: BMC Res Notes. 2009 Mar 23;2:46. doi: 10.1186/1756-0500-2-46 (PMC2674452; doi:10.1186/1756-0500-2-46)
Supplement: Additional File 5 — The sequences, targets and properties of the oligonucleotides used in this study are given. [file 1756-0500-2-46-S5.doc]

| **Use** | **Oligo** | **Target** | **Sequence 5' - 3'** | **Details** |
| --- | --- | --- | --- | --- |
| Amplifying pZJM insert | OL1724 | pZJMβ | CCCCAAGGGGTTATGCTAGT | For nested PCR of insert, sense |
|  | OL2056 | pZJMβ | TCGTATTAATCAGGTACCCCGG | Amplification/sequencing of insert, sense |
|  | OL2057 | pZJMβ | ATTCGATATCAAGCTTGGCC | Amplification/sequencing of insert, antisense |
|  |  |  |  |  |
| Amplifying gene fragments for tertiary screening | OL2211 | Tb927.5.3260 (hypothetical ORF) | AAGCTTGGTTACGTGGTTTCTGCTAAT | Amplification of fragment for RNAi (tertiary screen), sense |
|  | OL2212 | Tb927.5.3260 (hypothetical ORF) | GGATCCACACGTGAGAATGAGTACGG | Amplification of fragment for RNAi (tertiary screen), antisense |
|  | OL2215 | Tb10.6k15.2060 (TOR1) | AAGCTTAGGCAACCACTCGAGAATGC | Amplification of fragment for RNAi (tertiary screen), sense |
|  | OL2216 | Tb10.6k15.2060 (TOR1) | GGATCCAGTCCAGCATTATCGCTAAGA | Amplification of fragment for RNAi (tertiary screen), antisense |
|  | OL2219 | Tb927.3.930 (dynein heavy chain) | GTCAGGATCCCCAAGTTTGACAGAACGGGT | Amplification of fragment for RNAi (tertiary screen), sense |
|  | OL2220 | Tb927.3.930 (dynein heavy chain) | GGGAAGCTTCATAAGGAAGTGCAGTCGCA | Amplification of fragment for RNAi (tertiary screen), antisense |
|  | OL2321 | Tb927.1.1930 (TOR-like 2) | GTCAGGATCCAGACCTGAGCTTGTCGGAAA | Amplification of fragment for RNAi (tertiary screen), sense |
|  | OL2322 | Tb927.1.1930 (TOR-like 2) | GGGAAGCTTCTGCGTGAGCATAGGAAACA | Amplification of fragment for RNAi (tertiary screen), antisense |
|  | OL2715 | Tb11.01.0450 (PP1) | AACCAAGCTTCAACCGCATCTACGGTTTTT | Amplification of fragment for RNAi (tertiary screen), sense |
|  | OL2716 | Tb11.01.0450 (PP1) | AATTGGATCCACTCGTTGCAATAGTTGGGC | Amplification of fragment for RNAi (tertiary screen), antisense |
|  |  |  |  |  |
| Real Time PCR oligos | OL2272 | Tb10.61.3060 (*GPI8*) | CGAAGCGCATTTGGATAGC | Real time PCR, sense |
|  | OL2273 | Tb10.61.3060 (*GPI8*) | AGCGCGTGATGACAGTGAAG | Real time PCR, antisense |
|  | OL2494 | Tb10.6k15.2060 (TOR1) | GCGGAGACGTGGCTTGAG | Real time PCR, sense |
|  | OL2495 | Tb10.6k15.2060 (TOR1) | CAGTCCTAATATATGCCCCACCAT | Real time PCR, antisense |
|  | OL2552 | Tb927.8.3390 (hypothetical ORF) | ACCATCGGTGCTGAGACAATT | Real time PCR, sense |
|  | OL2553 | Tb927.8.3390 (hypothetical ORF) | CGGCTCTTCTCCATCGTTTT | Real time PCR, antisense |
|  | OL2554 | Tb927.8.3380 (electron transfer protein) | GGCGTTCCTTCAGATGATGAG | Real time PCR, sense |
|  | OL2555 | Tb927.8.3380 (electron transfer protein) | ACTGAAGAAGCGCCATCACA | Real time PCR, antisense |
|  | OL2556 | Tb11.01.0460 (hypothetical ORF) | CACATGAGACAGAGTGGCTTTCC | Real time PCR, sense |
|  | OL2557 | Tb11.01.0460 (hypothetical ORF) | CGAGACGCCGTGCAAAC | Real time PCR, antisense |
|  | OL2558 | Tb11.01.0450 (PP1) | CAACAGCGGTGCAGTAATGTCT | Real time PCR, sense |
|  | OL2559 | Tb11.01.0450 (PP1) | TTCTTTACGGATGGCTTAAGGATT | Real time PCR, antisense |
|  | PR64 | Tb927.1.1930 (TOR-like 2) | CGTTGAGCGGAATGGTAAGG | Real time PCR, sense |
|  | PR65 | Tb927.1.1930 (TOR-like 2) | GTTGGTATCACCCTCAATTGACTTG | Real time PCR, antisense |
